# Comparative performance of SARS-CoV-2 lateral flow antigen tests and association with detection of infectious virus in clinical specimens: a single-centre laboratory evaluation study

**DOI:** 10.1016/S2666-5247(21)00143-9

**Published:** 2021-09

**Authors:** Suzanne Pickering, Rahul Batra, Blair Merrick, Luke B Snell, Gaia Nebbia, Sam Douthwaite, Fiona Reid, Amita Patel, Mark Tan Kia Ik, Bindi Patel, Themoula Charalampous, Adela Alcolea-Medina, Maria Jose Lista, Penelope R Cliff, Emma Cunningham, Jane Mullen, Katie J Doores, Jonathan D Edgeworth, Michael H Malim, Stuart J D Neil, Rui Pedro Galão

**Affiliations:** a Department of Infectious Diseases, School of Immunology and Microbial Sciences, King's College London, London, UK; b Centre for Clinical Infection and Diagnostics Research, Department of Infectious Diseases, Guy's and St Thomas' NHS Foundation Trust, London, UK; c School of Population Health and Environmental Sciences, King's College London, London, UK; d Viapath Group, Guy's and St Thomas' NHS Foundation Trust, London, UK

## Abstract

**Background:**

Lateral flow devices (LFDs) for rapid antigen testing are set to become a cornerstone of SARS-CoV-2 mass community testing, although their reduced sensitivity compared with PCR has raised questions of how well they identify infectious cases. Understanding their capabilities and limitations is, therefore, essential for successful implementation. We evaluated six commercial LFDs and assessed their correlation with infectious virus culture and PCR cycle threshold (Ct) values.

**Methods:**

In a single-centre, laboratory evaluation study, we did a head-to-head comparison of six LFDs commercially available in the UK: Innova Rapid SARS-CoV-2 Antigen Test, Spring Healthcare SARS-CoV-2 Antigen Rapid Test Cassette, E25Bio Rapid Diagnostic Test, Encode SARS-CoV-2 Antigen Rapid Test Device, SureScreen COVID-19 Rapid Antigen Test Cassette, and SureScreen COVID-19 Rapid Fluorescence Antigen Test. We estimated the specificities and sensitivities of the LFDs using stored naso-oropharyngeal swabs collected at St Thomas' Hospital (London, UK) for routine diagnostic SARS-CoV-2 testing by real-time RT-PCR (RT-rtPCR). Swabs were from inpatients and outpatients from all departments of St Thomas' Hospital, and from health-care staff (all departments) and their household contacts. SARS-CoV-2-negative swabs from the same population (confirmed by RT-rtPCR) were used for comparative specificity determinations. All samples were collected between March 23 and Oct 27, 2020. We determined the limit of detection (LOD) for each test using viral plaque-forming units (PFUs) and viral RNA copy numbers of laboratory-grown SARS-CoV-2. Additionally, LFDs were selected to assess the correlation of antigen test result with RT-rtPCR Ct values and positive viral culture in Vero E6 cells. This analysis included longitudinal swabs from five infected inpatients with varying disease severities. Furthermore, the sensitivities of available LFDs were assessed in swabs (n=23; collected from Dec 4, 2020, to Jan 12, 2021) confirmed to be positive (RT-rtPCR and whole-genome sequencing) for the B.1.1.7 variant, which was the dominant genotype in the UK at the time of study completion.

**Findings:**

All LFDs showed high specificity (≥98·0%), except for the E25Bio test (86·0% [95% CI 77·9–99·9]), and most tests reliably detected 50 PFU/test (equivalent SARS-CoV-2 N gene Ct value of 23·7, or RNA copy number of 3 × 10^6^/mL). Sensitivities of the LFDs on clinical samples ranged from 65·0% (55·2–73·6) to 89·0% (81·4–93·8). These sensitivities increased to greater than 90% for samples with Ct values of lower than 25 for all tests except the SureScreen fluorescence (SureScreen-F) test. Positive virus culture was identified in 57 (40·4%) of 141 samples; 54 (94·7%) of the positive cultures were from swabs with Ct values lower than 25. Among the three LFDs selected for detailed comparisons (the tests with highest sensitivity [Innova], highest specificity [Encode], and alternative technology [SureScreen-F]), sensitivity of the LFDs increased to at least 94·7% when only including samples with detected viral growth. Longitudinal studies of RT-rtPCR-positive samples (tested with Innova, Encode, and both SureScreen-F and the SureScreen visual [SureScreen-V] test) showed that most of the tests identified all infectious samples as positive. Test performance (assessed for Innova and SureScreen-V) was not affected when reassessed on swabs positive for the UK variant B.1.1.7.

**Interpretation:**

In this comprehensive comparison of antigen LFDs and virus infectivity, we found a clear relationship between Ct values, quantitative culture of infectious virus, and antigen LFD positivity in clinical samples. Our data support regular testing of target groups with LFDs to supplement the current PCR testing capacity, which would help to rapidly identify infected individuals in situations in which they would otherwise go undetected.

**Funding:**

King's Together Rapid COVID-19, Medical Research Council, Wellcome Trust, Huo Family Foundation, UK Department of Health, National Institute for Health Research Comprehensive Biomedical Research Centre.

**Research in context**

**Evidence before this study**

We searched PubMed on April 22, 2021, with no date or language restrictions, using the terms (“SARS-CoV-2” OR “COVID-19”) AND (“antigen”) AND (“infectivity” OR “virus isolation”). Our search revealed 28 research publications, among which only two specifically addressed the characterisation of rapid antigen tests in the context of a correlation between their performance and sample infectivity in vitro. As evidence of a rapidly moving field, the same search in *medRxiv* identified several manuscripts showing either evaluations of different lateral flow devices (LFDs) for rapid antigen testing according to RT-PCR cycle threshold (Ct) values, or relationships between Ct values and virus infectivity. A common limitation of these papers was the scarcity of clear evidence of a relationship between antigen test positivity and the existence of infectious virus in the same clinical specimen. In addition, none of the studies reassessed the performance of rapid antigen tests in the context of longitudinal panels, or against the variant that was becoming dominant in the UK at the time of the study, B.1.1.7.

**Added value of this study**

This study is, to our knowledge, the largest to date assessing the correlation between Ct values, quantitative culture of infectious virus, and antigen test positivity, alongside an unbiased head-to-head comparison of six commercial antigen tests. We found that most rapid antigen tests performed to a high standard in clinical samples. Among three LFDs selected for detailed comparisons, we found a sensitivity of at least 94·7% when compared with samples that were infectious in vitro, with absolute viral titre in the specimens correlating with Ct values. Longitudinal studies of real-time-RT-PCR-positive samples provided evidence that differences in test sensitivities can lead to missed cases in the absence of repeated testing, which is particularly relevant in the context of asymptomatic or presymptomatic individuals. We also showed that despite amino acid changes in the SARS-CoV-2 nucleocapsid antigen, detection of the B.1.1.7 variant by selected LFD tests was not affected. This study provides clear evidence of the relationship between Ct values, cultivable virus, and antigen LFD positivity, with tests delivering reliable identification of infectious clinical samples.

**Implications of all the available evidence**

In a time when LFDs for rapid antigen testing are expected to have a major role in SARS-CoV-2 mass community and health-care testing, we believe that this study will inform the ongoing debate about how these tests should be deployed. Our data support regular testing of target groups with LFDs, not as standalone one-off tests, but rather to supplement current PCR testing capacity, and thus rapidly identify infectious individuals in situations in which they would otherwise go undetected.

## Introduction

COVID-19 continues to have a profound impact on global health, with many countries resorting to economically and socially damaging restrictions to minimise the spread of SARS-CoV-2 and protect health-care systems from being overwhelmed. Pathways out of national lockdowns, and strategies to mitigate the need for such measures in the future, depend on the successful implementation of mass vaccination programmes, effective contact tracing systems, and mass community testing. In addition to the existing PCR-based testing systems, mass community testing might take the form of targeted intensive testing in areas of increasing incidence, alongside regular routine screening in health-care, education, workplace, and leisure settings. Realistically, the expansion of regular testing relies on an element of low-infrastructure testing or self-testing, such as that offered by lateral flow devices (LFDs) for rapid antigen detection.^1^, ^2^

Thoroughly understanding the advantages and limitations of LFDs is, therefore, a priority, and will help to inform decisions about settings in which these tests will have the most utility and, conversely, those in which they could be contraindicated. There are concerns about the reduced sensitivity of LFDs in comparison with PCR, and controversies have emerged over the suitability of their implementation.^3^, ^4^, ^5^ Problems with comparing cycle threshold (Ct) values from RT-PCR between different protocols, and even between the same protocols at different locations, combined with uncertainty about the range of viral loads that constitute a transmission risk, have been the cause of many of the controversies.^5^, ^6^ Individuals are most infectious around the time of symptom onset, when viral loads in the upper respiratory tract are highest,^7^, ^8^ with recent studies confirming an association between viral load and increased transmission of SARS-CoV-2.^9^ For asymptomatic individuals, infectivity and viral load dynamics involve a similar, limited period of infectivity. Asymptomatic and presymptomatic contributions to viral spread in the community remain problematic, accounting for a notable proportion of transmissions but often going undetected.^10^, ^11^

Several studies have shown a relationship between Ct value and culture of infectious virus,^8^, ^12^, ^13^, ^14^ and manufacturers of LFDs have implied a link between antigen test positivity and infectious potential. The aim of this study was to assess in detail the relationship between Ct value, viral load, quantitative culture of infectious virus, and antigen test positivity, and provide an independent and unbiased head-to-head comparison of six widely available commercial antigen tests. Tests were also reassessed in consideration of the emergence and spread of the SARS-CoV-2 B.1.1.7 genotype in the UK, first detected in November, 2020.

## Methods

### Study samples

In a single-centre, laboratory evaluation study, we evaluated the performance of six rapid antigen tests commercially available in the UK by head-to-head comparison. Combined naso-oropharyngeal swabs were submitted for routine diagnostic SARS-CoV-2 testing by real-time RT-PCR (RT-rtPCR) to the Viapath Infection Sciences Laboratory (St Thomas' Hospital, London, UK) in 1 mL of viral transport medium (VTM; Sigma Virocult, Medical Wire & Equipment, Corsham, UK). Surplus VTM was stored immediately at –80°C after determination of a diagnostic result, with no additional freeze-thaw cycles before inclusion in the study. Swabs processed by the laboratory were from inpatients and outpatients from all departments of St Thomas' Hospital, and from health-care staff across all departments and their household contacts (a breakdown of the numbers of inpatients, outpatients, staff, and contacts is not available due to masking of the research team to this information).

VTM from 100 SARS-CoV-2-negative swabs (confirmed by RT-rtPCR) from the same population were used for comparative specificity determinations. Two different SARS-CoV-2-positive sample sets (RT-rtPCR-confirmed) were used: one set for head-to-head sensitivity comparisons of six commercial antigen tests (n=100) and one set for comparative studies of infectivity and antigen test positivity (n=141). A single sample set could not be used for all analyses because the volume of VTM required exceeded that provided by a single sample; therefore, two collections of samples were used. There was no difference in how these samples were selected. All samples were collected between March 23 and Oct 27, 2020, and were demographically representative of the typical population providing samples for testing at the diagnostic laboratory during this period. Confirmed positive samples were selected to cover a wide range of Ct values (12·7 to 40·0), but were not subjected to any further selection criteria. Sampling time from symptom onset ranged from –1 to 37 days. An independent subset of sequential swabs from five inpatients with different disease severities (two asymptomatic, two mild, and one severe; whereby we planned to include different disease severities within the constraints of sample availability, with details of disease scoring reported previously^15^, ^16^), collected as part of the patient's routine standard of care, were used for longitudinal studies of infectivity and antigen test positivity (between two and five longitudinal samples per individual; 21 samples overall). A further 23 RT-rtPCR-confirmed SARS-CoV-2 positive swabs, collected from Dec 4, 2020, to Jan 12, 2021, were shown by on-site whole-genome sequencing (Oxford Nanopore Technologies, Oxford, UK) to be from the B.1.1.7 variant and used for comparative evaluation of the sensitivity of available LFDs (based on kit availability). These were compared with samples from April and September, 2020 (n=23 for each month; assumed to be non-B.1.1.7 variant) from the SARS-CoV-2-positive sample sets, which were selected on the basis of approximately equivalent Ct values to the B.1.1.7 samples. To minimise sample deterioration, all VTM samples were thawed once and immediately subjected to RNA extraction (for confirmatory RT-rtPCR), LFD assessment, and viral growth assays as appropriate.

### RT-rtPCR

Initial diagnostic laboratory testing was done with the AusDiagnostics multiplexed-tandem PCR assay including SARS-CoV-2 (Chesham, UK), and positive and negative swabs were selected on the basis of this diagnostic test. For confirmatory PCR testing and to ensure uniformity of RT-rtPCR conditions and Ct determination, RNA was extracted from 100 μL swab with the Qiagen QIAamp Viral RNA Kit (Hilden, Germany) following manufacturer's instructions and eluted in 60 μL water. RT-rtPCR reactions (total volume 20 μL) were done with 5 μL eluted RNA, TaqMan Fast Virus 1-Step Master Mix (4X formulation; Applied Biosystems, Waltham, MA, USA), and primer-probes sets targeting SARS-CoV-2 nucleocapsid (SARS-CoV-2-N; N1 set) gene regions or human RNAse P designed by the US Centers for Disease Control and Prevention (manufactured by Integrated DNA Technologies, Coralville, IA, USA), with a QuantStudio 5 Real-Time PCR System (ThermoFisher Scientific, Waltham, MA, USA). For the calculation of viral loads (RNA copies per mL), RNA standards were extracted as described from serial dilutions of a NATtrol SARS-CoV-2 Stock (ZeptoMetrix, Buffalo, NY, USA), which is formulated with purified, inactivated, intact viral particles of known RNA copies per /mL. A calibration step was used to determine SARS-CoV-2-N Ct value (appendix p 3; referred to as N Ct value hereafter).

### Rapid antigen tests

The following SARS-CoV-2 rapid antigen tests were used for comparative studies: Innova Rapid SARS-CoV-2 Antigen Test (Xiamen Biotime Biotechnology, Fujian, China), Spring Healthcare SARS-CoV-2 Antigen Rapid Test Cassette (Shanghai ZJ Bio-Tech, Shanghai, China), E25Bio Rapid Diagnostic Test (E25Bio, Cambridge, MA, USA), Encode SARS-CoV-2 Antigen Rapid Test Device (Zhuhai Encode Medical Engineering, Zhuhai, China), SureScreen COVID-19 Rapid Antigen Test Cassette, and SureScreen COVID-19 Rapid Fluorescence Antigen Test (both from SureScreen Diagnostics, Derby, UK). We refer to the test kits as Innova, Spring Healthcare, E25Bio, Encode, SureScreen visual (SureScreen-V), and SureScreen fluorescent (SureScreen-F) hereafter. To allow extensive comparative studies alongside the determination of infectious virus in clinical samples, studies were done on swabs stored in VTM, rather than direct swabs taken immediately before tests. 50 μL of stored VTM was mixed with 100 μL buffer supplied by each test kit, and 100 μL of this solution was applied to the test cassette, as per manufacturer instructions. To identify the limit of detection (LOD) for each test, known plaque-forming units (PFUs) of SARS-CoV-2 (England 02/2020 strain; Public Health England, Porton Down, UK) were propagated in Vero E6 cells and diluted in phosphate-buffered saline, and 50 μL of each dilution was mixed with 100 μL kit-supplied buffer. 100 μL of this solution was applied to each test, equating to 1–10 000 PFUs/test or 30–300 000 PFU/mL, and each quantity was tested in triplicate for all tests. NATrol SARS-CoV-2 Stock (ZeptoMetrix, Buffalo, NY, USA) was tested as a post-hoc control on tests with the best LOD. Results of all tests were read at the time instructed by the manufacturer (between 10 and 30 min); results were recorded independently by two readers (SP and RPG) and compared, and in the event of a discordant reading referred to a third individual (RB or MTKI). For purposes of comparison, the chromatographic tests were scored according to whether the test band was strongly positive, unequivocally positive, weakly positive, or negative, with the exception of the SureScreen-F test, which is machine-read and delivers a binary result (positive or negative). Test band scoring was used to provide detailed information on the nature of the test result, but all sensitivity and specificity calculations were based on the binary results of the tests. Details of the tests and examples of the scoring of tests are given in the appendix (pp 4, 7). Although the tests are qualitative and all results were treated as binary, results are also displayed as a heatmap (appendix p 7) to convey the magnitude of the result, allowing more detailed comparisons between the tests and potentially informing future use.

### Viral growth assays

For the comparative studies of infectivity and antigen positivity, each swab was subjected to the following procedures: RNA extraction for subsequent RT-rtPCR and sequencing; titration and viral titre measurement by plaque assay; titration and infectivity determination by intracellular anti-SARS-CoV-2-N staining (in samples with sufficient volume remaining); viral propagation for isolation of virus; and LFD testing (appendix p 3). LFDs were selected for the correlative study of infectivity and antigen test positivity on the basis of highest sensitivity and highest specificity (in the initial comparative studies), and use of an alternative technology (SureScreen-F). However, due to sample volume or test availability, not all samples could be assayed in all selected tests. To avoid bias due to comparing different samples, results were also analysed for the subset of samples available for all tests. Viral growth assays were done in Vero E6 cells (appendix p 3).

### Statistical analysis

Linear regressions and associated R^2^ and p values were determined to test the relationship between observed Ct values for the N gene and either log_10_ SARS-CoV-2 viral load (measured as RNA copies per mL) or log_10_ SARS-CoV-2 PFUs/ml. Exact binomial 95% CIs for the specificity and sensitivity of all LFDs were determined with the Wilson–Brown method. Analytical sensitivities in clinical samples were also assessed with binomial logistic regressions fitted to a binary dependent Y variable (LFD or viral culture), and an independent X variable (Ct N value or log_10_ SARS-CoV-2 viral load measured as RNA copies per mL). These regression models were used to determine 50% detection rates (and corresponding 95% CIs) of the LFDs, defined as the predicted Ct N or RNA viral load concentrations measured by RT-rtPCR at which 50% of results were positive in each LFD or for viral culture. A receiver operating characteristic curve and corresponding area under the curve were determined for each logistic regression. Cumulative sensitivity was calculated for each LFD across ascending Ct values in single-sample increments. Sensitivities and specificities were compared for paired samples across test kits with McNemar's test (head-to-head comparisons). Fisher's exact test was used to compare sensitivities for B.1.1.7 variant swabs with earlier variant specimens collected in 2020. All tests were two-sided, and p values lower than 0·05 were considered statistically significant. All statistical analyses were calculated in GraphPad Prism 9.

### Role of the funding source

The funder of the study had no role in study design, data collection, data analysis, data interpretation, or writing of the report, or in the decision to submit this manuscript for publication.

## Results

Six commercial rapid antigen tests (Innova, E25Bio, Spring Healthcare, Encode, SureScreen-V, and SureScreen-F; appendix p 4) were compared for specificity, LODs, and sensitivity.

Specificity was determined for each test with a panel of 100 RT-rtPCR-confirmed SARS-CoV-2-negative swabs (table 1). All tests showed high (≥98·0%) specificity, with the exception of E25Bio (86·0% [95% CI 77·9–99·9]). SureScreen-V and Encode both achieved a specificity of 100·0% (96·3–100·0). None of the negative samples gave a false-positive result for more than one test kit, suggesting that false positives appear stochastically and are not a particular feature of the samples. All false positives were only weakly positive, with the exception of SureScreen-F, for which this information was not available as the electronic reader delivers a binary result.

**Table 1:** Specificity and sensitivity of six commercial SARS-CoV-2 rapid antigen tests compared with real-time RT-PCR

	**Number of samples**	**Specificity (95% CI)**	**Number of samples**	**Sensitivity (95% CI)**	**RNA copies per mL at 50% detection rate (95% CI)**	**N Ct value at 50% detection rate (95% CI)**	**ROC AUC (95% CI)**
**Innova**							
Overall	100	99·0% (94·6–99·9)	100	89·0% (81·4–93·8)	2·29 × 10^4^ (1·70 × 10^3^–9·55 × 10^4^)	30·7 (28·8–34·3)	0·948 (0·896–0·999)
N Ct value <28	..	..	88	95·5% (88·9–98·2)	..	..	..
N Ct value <25	..	..	69	98·6% (92·2–99·9)	..	..	..
**Spring Healthcare**							
Overall	100	98·0% (93·0–99·6)	100	77·0% (67·8–84·2)	3·8 × 10^5^ (1·35 × 10^5^–8·51 × 10^5^)	26·8 (25·8–28·3)	0·949 (0·908–0·989)
N Ct value <28	..	..	88	85·2% (76·4–91·2)	..	..	..
N Ct value <25	..	..	69	95·7% (88·0–98·8)	..	..	..
**E25Bio**							
Overall	100	86·0% (77·9–99·9)	100	75·0% (65·7–82·4)	4·27 × 10^5^ (1·26 × 10^5^–1·07 × 10^6^)	26·7 (25·5–28·4)	0·915 (0·854–0·975)
N Ct value <28	..	..	88	83·0% (73·8–89·4)	..	..	..
N Ct value <25	..	..	69	94·2% (86·0–97·7)	..	..	..
**Encode**							
Overall	100	100·0% (96·3–100·0)	100	74·0% (64·6–81·6)	6·31 × 10^5^ (2·69 × 10^5^–1·29 × 10^6^)	26·2 (25·2–27·4)	0·956 (0·920–0·992)
N Ct value <28	..	..	88	83·0% (73·8–89·4)	..	..	..
N Ct value <25	..	..	69	94·2% (86·0–97·7)	..	..	..
**SureScreen-F**							
Overall	100	98·0% (93·0–99·6)	100	69·0% (59·4–77·2)	8·91 × 10^5^ (2·57 × 10^5^–2·24 × 10^6^)	25·7 (24·4–27·4)	0·891 (0·819–0·962)
N Ct value <28	..	..	88	75·0% (65·0–82·9)	..	..	..
N Ct value <25	..	..	69	88·4% (78·8–94·0)	..	..	..
**SureScreen-V**							
Overall	100	100·0% (96·3–100·0)	100	65·0% (55·2–73·6)	2·09 × 10^6^ (1·12 × 10^6^–3·80 × 10^6^)	24·5 (23·7–25·4)	0·968 (0·936–1·000)
N Ct value <28	..	..	88	73·9% (63·8–81·9)	..	..	..
N Ct value <25	..	..	69	91·3% (82·3–96·0)	..	..	..

**Figure 1:**
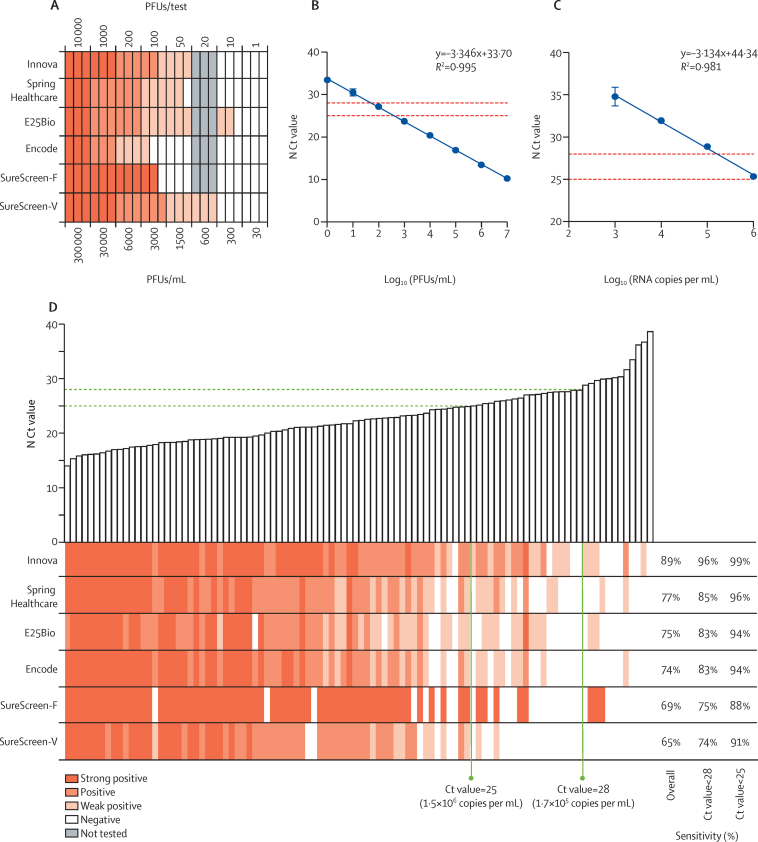
Comparative sensitivity of six commercial SARS-CoV-2 rapid antigen tests (A) Heatmap comparison of lower limit of detection. (B) Association between PFUs/mL and Ct result from RT-rtPCR for the SARS-CoV-2 N gene (N Ct value). Error bars represent SD of three independent experiments. (C) Association between RNA copy number and N Ct values. Copy number per mL was derived from N RT-rtPCR on the Zeptometrix RNA standard (consisting of purified, inactivated viral particles of known RNA copy number per mL). In (B) and (C), points were fitted with a linear regression, with the equation of the line and the *R*^2^ value shown. Horizontal dashed lines denote Ct values of 25 and 28, as the threshold cutoffs used for sensitivity determinations. (D) Tests were evaluated in head-to-head comparisons on a panel of 100 SARS-CoV-2-positive naso-oropharyngeal swabs. Bars denote the N Ct result for each swab, in ascending order, with the antigen test results for each sample directly below each bar presented as a heatmap. Sensitivity determinations from this sample set are shown on the right. Ct value cutoffs of 25 and 28, corresponding to 1·49 × 10^6^ and 1·65 × 10^5^ RNA copies per mL, or 400 and 50 PFUs/mL, respectively, are indicated, with corresponding sensitivity values for each test at each threshold on the right. PFU=plaque-forming unit. SureScreen-F=SureScreen fluorescent. SureScreen-V=SureScreen visual. N=SARS-CoV-2 nucleocapsid. Ct=cycle threshold. RT-rtPCR=real-time RT-PCR.

ROC curves (not shown) and corresponding AUCs were determined in logistic regressions. A Ct value of 28 (corresponding to 50 PFUs/ml) is the approximate value at which all the tests failed to detect our viral stock dilutions (figure 1A); a Ct value of 25 provided a 10-times interval from Ct=28, and was chosen to facilitate the data analysis and highlight the observation that test sensitivities are higher at lower Ct values. SureScreen-F=SureScreen fluorescent. SureScreen-V=SureScreen visual. N=SARS-CoV-2 nucleocapsid. Ct=cycle threshold. ROC=receiver operating characteristic. AUC=area under the curve.

LODs were determined with specified PFUs of SARS-CoV-2 propagated in Vero E6 cells, applied to each test in triplicate. Informed by the specificity determinations, in which we observed very few false positives, any visible band was considered positive regardless of intensity or relationship to the control band. Most tests reliably detected 50 PFUs/test (1500 PFUs/mL) with the exception of Encode and SureScreen-F (figure 1A). SureScreen-V and Innova had the lowest consistent LOD (excluding E25Bio due to poor specificity), and on further testing of SureScreen-V, this test also consistently detected 20 PFUs/test (600 PFUs/mL). Calibration experiments with SARS-CoV-2 laboratory stock (figure 1B) and standardised RNA control reagents (figure 1C) delivered the equivalent N gene Ct value of 23·7 or RNA copy number of 3x10^6^/mL for the LOD of 1500 PFUs/mL. As particle-to-infectious unit ratios can vary between viral variants or according to growth or assaying conditions, as a post-hoc control we applied the Zeptometrix NATrol inactivated viral particle standard to Innova and SureScreen-V, as the two tests with the best LOD. This standard showed as weakly positive on both tests at 1·2 × 10^6^ RNA copies per mL, or projected Ct value of 25, in agreement with the results shown in figure 1.

Sensitivity comparisons on clinical samples were done as head-to-head evaluations on 100 SARS-CoV-2-positive combined naso-oropharyngeal swabs with Ct values ranging from 14·0 to 39·0 (figure 1D, appendix p 8). The 50% detection rates and cumulative sensitivities across all Ct values are shown in the appendix (p 8). Innova had the highest overall sensitivity (89·0% [95% CI 81·4–93·8]) for the clinical samples, with this increasing to 95·5% (88·9–98·2) when applied to samples with Ct values lower than 28, and 98·6% (92·2–99·9) when applied to samples with Ct values lower than 25 (table 1, appendix p 8). All other tests had overall sensitivities of between 65·0% and 77·0%, increasing to greater than 90% for samples with Ct values lower than 25 for all tests except SureScreen-F. Thus, we found good sensitivity and specificity for all tests on swabs within a defined Ct value window.

**Figure 2:**
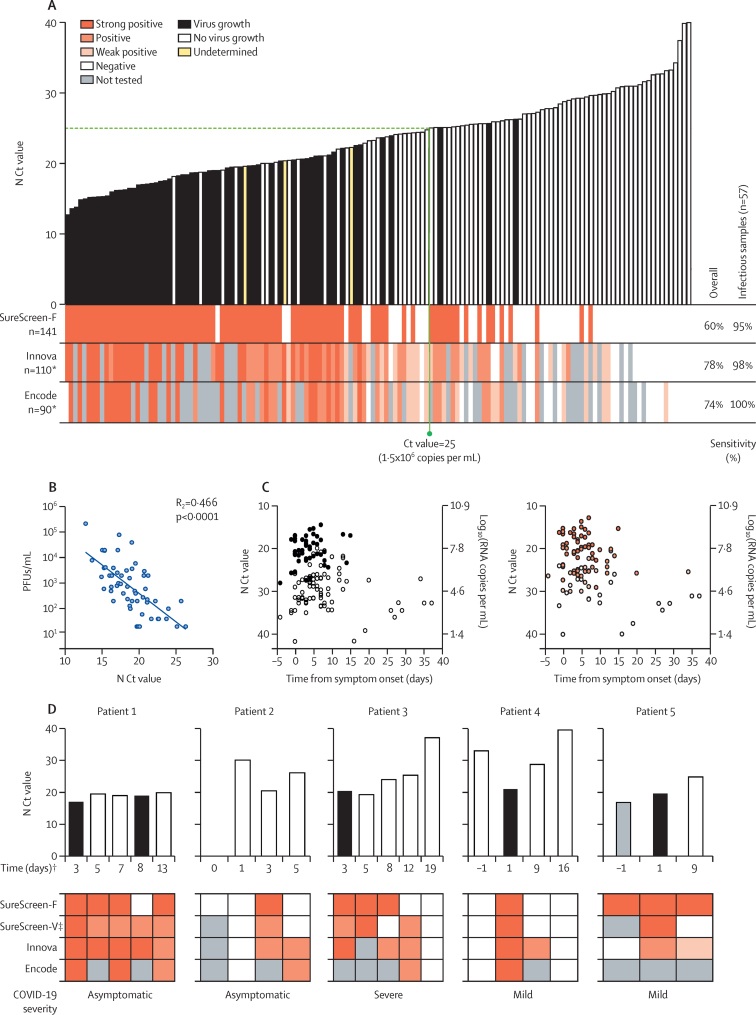
Comparison of Ct value, infectivity, and rapid antigen test result (A) Infectivity, N Ct value, and antigen test results for three commercial tests. Bars show the N Ct result for each swab in ascending order, coloured according to whether virus was cultured from the sample. Antigen test results for each sample are shown below each bar. Sensitivity determinations for the sample set are shown on the right. (B) Direct viral titres of swabs determined by plaque assay and compared with N Ct value. The linear relationship between Ct value and log_10_ concentration of infectious virus in 57 samples is shown. (C) Ct results from (A) and RNA copies per mL plotted against days from symptom onset. Points are coloured according to virus growth (left graph; n=141) or the score derived from the Innova antigen test (right graph; n=110). (D) Longitudinal examples of infectivity, antigen test positivity, and Ct values for five infected individuals with varying COVID-19 severities.^15^, ^16^ Bars show the N Ct value for each sample, shaded according to virus culture result. Antigen test results for each sample are shown below the bars. N=SARS-CoV-2 nucleocapsid. Ct=cycle threshold. SureScreen-F=SureScreen fluorescent. SureScreen-V=SureScreen visual. *Less than total sample due to limited sample volume or number of test kits available. †Time post-onset of symptoms for symptomatic individuals; time from the first positive PCR test for asymptomatic individuals. ‡Sufficient sample volume and test availability for inclusion in the longitudinal study for completeness of the dataset.

Three of the rapid antigen tests from the first phase of comparisons were selected for more detailed comparisons: the tests with highest sensitivity (Innova), highest specificity (Encode), and alternative technology (fluorescent machine-read result; SureScreen-F). 141 combined naso-oropharyngeal swabs were compared for N Ct value, antigen test result, and positive viral culture (figure 2A). Samples covered a range of Ct values (12·7 to 40·0). The direct viral titre of the swabs was determined by plaque assay of serially diluted swabs, with additional confirmatory intracellular anti-SARS-CoV-2 nucleocapsid staining performed on viral culture samples for 110 samples of sufficient volume. 57 (40·4%) of the 141 samples were positive for viral growth. 54 (94·7%) of the 57 cultures positive for viral growth had Ct values lower than 25, and the highest Ct value from a sample with positive viral culture was 26·3. The latest timepoint that virus was isolated was 15 days after symptom onset. Titres of infectious virus in the samples showed a moderate inverse linear relationship with N Ct values (R^2^=0·47, p<0·0001; figure 2B). Both viral culture and antigen test positivity were associated with Ct value, rather than the timing of the sample relative to symptom onset (figure 2C, appendix p 9).

Among the three selected antigen tests, overall test sensitivities and rankings were similar to those observed in initial assessments (figure 1), with Innova providing the highest sensitivity at 78·2% (95% CI 69·6–84·9), followed by Encode (74·4% [64·6–82·3]) and SureScreen-F (60·3% [52·0–68·0]; table 2, appendix p 8). When compared only with the samples from which virus was cultured, all tests achieved a sensitivity of at least 94·7% (figure 2A, table 2). For the subset of available samples tested on all three LFDs (n=90 for sensitivity, n=34 for infectious samples) to give a head-to-head comparison, we observed similar results to the overall comparison (table 2, appendix p 5).

**Table 2:** Sensitivity of three commercial SARS-CoV-2 rapid antigen tests compared with RT-rtPCR and virus isolation

		**Number of samples**	**Sensitivity *vs* RT-rtPCR (95% CI)**	**RNA copies per mL at 50% detection rate (95% CI)**	**N Ct value at 50% detection rate (95% CI)**	**ROC AUC (95% CI)**	**Number of samples**	**Sensitivity *vs* virus isolation (95% CI)**
**Encode**								
Overall		90†	74·4% (64·6–82·3)	1·31 × 10^5^ (3·47 × 10^4^–3·71 × 10^5^)	28·3 (26·9– 30·1)	0·931 (0·879–0·984)	34	100·0% (89·8–100·0)
	N Ct value <28	70	88·6% (79·0–94·1)	..	..	..	..	..
	N Ct value <25	52	96·2% (87·0–99·3)	..	..	..	..	..
Subset*		90	74·4% (64·6–82·3)	..	..	..	..	..
	N Ct value <28	70	96·2% (87·0–99·3)	..	..	..	..	..
	N Ct value <25	52	88·6% (79·0–94·1)	..	..	..	..	..
**Innova**								
Overall		110†	78·2% (69·6–84·9)	1·31 × 10^5^ (3·47 × 10^4^–3·71 × 10^5^)	28·3 (26·9–30·1)	0·931 (0·879–0·984)	46	97·8% (88·7–99·9)
	N Ct value <28	88	92·0% (84·3–96·0)	..	..	..	..	..
	N Ct value <25	66	97·0% (89·6–99·5)	..	..	..	..	..
Subset*		90	76·7% (67·0–84·2)	1·10 × 10^5^ (3·31 × 10^4^–2·82 × 10^5^)	28·6 (27·3–30·2)	0·957 (0·919–0·996)	34	100·0% (89·8–100·0)
	N Ct value <28	70	91·4% (82·5–96·0)	..	..	..	..	..
	N Ct value <25	52	98·1% (89·9–99·9)	..	..	..	..	..
**SureScreen-F**								
Overall		141	60·3% (52·0–68·0)	1·38 × 10^6^ (6·31 × 10^5^–2·88 × 10^6^)	25·1 (24·1–26·2)	0·918 (0·875–0·962)	57	94·7% (85·6–98·6)
	N Ct value <28	111	74·8% (66·0–81·9)	..	..	..	..	..
	N Ct value <25	82	84·2% (74·7–90·5)	..	..	..	..	..
Subset*		90	55·6% (45·3–65·4)	2·04 × 10^6^ (8·12 × 10^5^–4·68 × 10^6^)	24·6 (23·4–25·8)	0·918 (0·864–0·971)	34	100·0% (89·8–100·0)
	N Ct value <28	70	71·4% (60·0–80·7)	..	..	..	..	..
	N Ct value <25	52	78·8% (66·0–87·8)	..	..	..	..	..

SureScreen-F=SureScreen fluorescent. RT-rtPCR=real-time RT-PCR. N=SARS-CoV-2 nucleocapsid. Ct=cycle threshold. ROC=receiver operating characteristic. AUC=area under the curve.

* Test results were re-analysed due to differences in sample number between Encode, Innova, and SureScreen-F (for the subset of samples available for all three tests: n=90 for sensitivity compared with RT-rtPCR, n=34 for sensitivity compared with virus isolation); two-tailed p values for these subsets, calculated with McNemar's test for all possible permutations, are shown in the appendix (p 5).

† Due to insufficient sample volume or test unavailability, not all samples could be assayed on Innova and Encode.

To investigate how antigen test result changed with time, preliminary investigations were done on sequential naso-oropharyngeal swabs from five infected inpatients with varying disease severities (appendix p 6). Samples were compared for N Ct value, antigen test result (SureScreen-F, SureScreen-V, Innova, and Encode), and positive viral culture (figure 2D). All tests identified infectious samples as positive (with the exception of one sample tested by SureScreen-F), and most tests in most patients continued to deliver positive results for several days after peak infectivity. In patients 2, 3, and 4, Innova (and when available, Encode) tested positive for several days longer than SureScreen-F, although the exact length of this extended positivity cannot be stated as intermediate samples were not obtained. In two patients, RT-rtPCR testing identified preinfectious individuals (patients 2 and 4), who were negative in all antigen tests at the time of initial RT-rtPCR testing. 2 days later, a drop in Ct value in patient 4 coincided with antigen test positivity for all tests and the isolation of infectious virus. The longitudinal results in five individuals (figure 2D), together with our plots of N Ct result and viral culture against days from symptom onset in the wider sample (56 individuals with a determinate culture result across a range of timepoints; figure 2C), highlight the importance of repeat testing, rather than one-off testing, with LFD rapid antigen tests.

**Figure 3:**
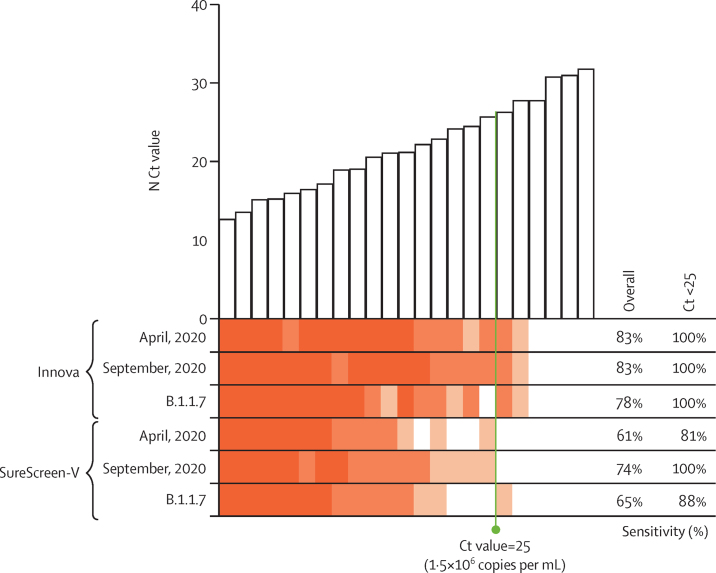
Comparative evaluation of antigen test sensitivity for the B.1.1.7 variant versus non-B.1.1.7 variant Combined naso-oropharyngeal swabs were obtained from 23 individuals with confirmed SARS-CoV-2 B.1.1.7 infection (December, 2020, and January, 2021) and compared with samples from before the variant was widely circulating in the UK population (April and September, 2020). All swabs were matched for N Ct values, shown in ascending order in the graph, and tested on Innova and SureScreen-V rapid antigen tests. N=SARS-CoV-2 nucleocapsid. Ct=cycle threshold. SureScreen-V=SureScreen visual.

Given that the rapid antigen tests rely on antibody detection of SARS-CoV-2-N, even single amino acid mutations have the potential to affect test sensitivity. As such, test performance should be reassessed for new emerging variants of SARS-CoV-2, such as B.1.1.7, which by the end of our study had become the dominant genotype in the UK, and contains four mutations in SARS-CoV-2-N compared with the England 02/2020 strain (Asp3Leu, Arg203Lys, Gly204Arg, and Ser235Phe).^17^ We did small-scale evaluations using swab samples spanning a range of Ct values (12·7 to 31·8), from April and September, 2020, and B.1.1.7-positive swabs (confirmed by viral sequencing) between December, 2020, and January, 2021 (figure 3). Both Innova and SureScreen-V tests showed variations in sensitivities between the three batches of samples tested, as would be expected for biological samples, but test results were qualitatively similar, with no evidence of altered sensitivity for the B.1.1.7 swabs (table 3).

**Table 3:** Sensitivity comparisons of two commercial SARS-CoV-2 rapid antigen tests on B.1.1.7 samples *vs* samples from April and September 2020

	**Innova**			**SureScreen-V**		
	Number of samples	Sensitivity *vs* RT-rtPCR (95% CI)	p value*	Number of samples	Sensitivity *vs* RT-rtPCR (95% CI)	p value*
**B.1.1.7**						
Overall	23	78·3% (58·1–90·3)	..	23	65·2% (44·9–81·2)	..
N Ct value <25	16	100·0% (80·6–100)	..	16	87·5% (64·0–97·8)	..
**April, 2020**						
Overall	23	82·6% (62·9–93·0)	1·0	23	60·9% (40·8–77·8)	1·0
N Ct value <25	16	100·0% (80·6–100)	1·0	16	81·2% (57·0–93·4)	1·0
**September, 2020**						
Overall	23	82·6% (62·9–93·0)	1·0	23	73·9% (53·5–87·4)	0·75
N Ct value <25	16	100·0% (80·6–100·0)	1·0	16	100·0% (80·6–100·0)	0·48

RT-rtPCR=quantitative RT-PCR. N=SARS-CoV-2 nucleocapsid. Ct=cycle threshold. SureScreen-V=SureScreen visual.

* Two-tailed p values were determined for the comparison of sensitivities between the B.1.1.7 samples (December, 2020, and January, 2021) and earlier samples (assumed to be non-B.1.1.7) with Fischer's exact test.

## Discussion

By extensive head-to-head comparison, we found that most rapid antigen tests performed to a high standard, with good sensitivity and excellent specificity. Consistent with previous reports, the tests delivered an overall sensitivity of 65·0–89·0% in comparison with RT-rtPCR.^18^, ^19^, ^20^, ^21^ increasing to greater than 90% for most tests for samples with Ct values of less than 25.^19^, ^22^, ^23^ Among the Innova, Encode, and SureScreen-F tests, sensitivity increased to at least 95% when compared with samples that were infectious in vitro, with viral titre in the specimens correlating with Ct values. This study, to our knowledge, provides the most comprehensive comparison of antigen LFDs and infectivity to date, showing a clear relationship between Ct values, quantitative culture of infectious virus, and antigen LFD positivity, with the tests used in each analysis delivering reliable identification of infectious clinical samples. The positive samples included in this study were representative of those encountered by the diagnostic laboratory at St Thomas' Hospital, including from health-care workers and their household contacts, asymptomatic individuals undergoing unrelated hospital treatment, and individuals with mild to severe COVID-19. Given the breadth of samples, we expect the findings from this study to apply to a wide range of infection scenarios.

In agreement with previous studies, we cultured virus from upper respiratory tract specimens with Ct values of up to 26 (an equivalent viral load of 7 × 10^5^ RNA copies per mL), with most of the culturable samples taken in the first week after symptom onset.^12^, ^13^, ^14^, ^24^, ^25^ The minimum viral titre required for transmission is unclear^25^ and will depend in part on the proximity and duration of contact. Nevertheless, higher viral loads, as measured by lower Ct, have been strongly associated with transmission,^9^ and, therefore, a reasonable assumption is that the quantity of cultured virus in vitro has a similar correlation with infectivity. However, problems arise when attempting to standardise Ct values as surrogate measures of transmission potential, due to differences in RNA extraction and RT-PCR methods. This issue was indicated in a recent study,^4^ in which differences in Ct value of greater than 5 between RT-PCR systems prompted concerns that LFDs were missing up to 50% of potentially infectious cases.^4^, ^5^, ^6^ There have been frequent suggestions for SARS-CoV-2 results to be presented as viral load (RNA copies per mL) due to difficulties in comparing Ct values between studies. However, the absence of an agreed standard for determining viral load is itself a problem, with reported viral loads often appearing even more disparate than Ct values.^26^

Although the reduced sensitivity of LFDs relative to PCR is less of a concern late in the infection course when Ct values are increasing and the risk of onward transmission is negligible,^9^ reduced sensitivity can be problematic during the presymptomatic or early asymptomatic phase of infection.^10^ As shown by our longitudinal studies and our data suggesting early timepoints with higher Ct values give negative results with LFD, an individual can be positive by PCR but negative according to antigen testing for 1 or 2 days before testing positive. A negative result delivered at this stage in the course of infection could offer false security to someone who is about to become highly infectious. Furthermore, with the time window of positive results narrower than for PCR testing, relatively small differences in test sensitivities could translate to capturing or missing potentially infectious cases. We, therefore, recommend that regular testing be emphasised, and that tests are deployed in populations in which the limitations of these tests are understood and manageable.

In particular inpatient situations, LFDs can also be used to make early, rapid decisions about patient management, with appropriate isolation pending confirmatory PCR testing. This approach has been successful in hospital LFD pilot studies, with the use of such devices preventing the cohorting of asymptomatic and infectious individuals with uninfected patients while awaiting PCR results (unpublished). Towards the end of the disease course, LFDs could also be useful for determining if persistently PCR-positive individuals pose a transmission risk, potentially in tandem with rapid antibody testing.^27^

Although the LFDs are easy to use, the correct sampling, reading, and interpretation of the result are essential to their success in mass screening situations.^1^ In particular, mass screening programmes need to consider training and familiarity with swabbing when deploying devices to the general public as compared with a trained health-care worker in a hospital or clinical setting. It is also easy to underestimate the importance of correctly recognising a positive band. We found that some tests gave clearer results than others, which was the rationale for presenting our results as heatmaps, and is illustrated by the number of weak positive results we observed. Removing this element of subjectivity, for example with use of a smartphone application to read or capture the LFD result, could improve success rates.

Our comparative studies were done with VTM-stored swabs used for routine SARS-CoV-2 diagnostic testing, to accurately reflect the broad range of typical cases encountered and to conduct the detailed viral infectivity studies that were central to this study. However, the LFDs are intended to be used with direct swabs and, therefore, our approach introduced a predilution step, and potentially underestimated the sensitivity of the LFDs. Conversely, self-administration of swabs in community testing could also affect sensitivity. Future studies would benefit from a direct comparison between self-administered dry swabs, VTM-stored swabs, and RT-PCR.

A further point for consideration is the need for continued reassessment of LFDs in the context of emerging new variants of SARS-CoV-2. Most LFDs use monoclonal antibodies targeting the SARS-CoV-2-N protein, and amino acid mutations in the N protein have been documented for notable variants, such as those first identified in the UK (B.1.1.7), Brazil (P1), South Africa (B.1.351), and India (B.1.617).^28^ With the potential for accumulating mutations to occur in emerging variants, alongside possible differences in infectivity and tranmissibility, consistency in sensitivity for a given test should not be assumed.

Our data support the judicious use of LFDs for rapid antigen detection: not to replace PCR testing, but to supplement current testing capacity and rapidly identify infected individuals in situations in which they would otherwise go undetected. Although sensitivity is lower than with PCR-based testing, the rapid turnaround of these LFD tests, their versatility in terms of cost and portability, and their utility in disrupting transmission chains^2^, ^29^ originating from infectious asymptomatic individuals could outweigh the risk of missing positive cases.

## Data sharing

All relevant data are within the manuscript and appendix.

## Declaration of interests

We declare no competing interests.
