# Correction to *Lancet Microbe* 2021; published online June 29. https://doi.org/10.1016/S2666-5247(21)00143-9

**DOI:** 10.1016/S2666-5247(21)00210-X

**Published:** 2021-09

*Pickering S, Batra R, Merrick B, et al. Comparative performance of SARS-CoV-2 lateral flow antigen tests and association with detection of infectious virus in clinical specimens: a single-centre laboratory evaluation study.* Lancet Microbe *2021; published online June 30. https://doi.org/10.1016/ S2666-5247(21)00143-9*—This Article should have been published under a CC BY Open Access license. This correction has been made as of Aug 3, 2021.

