# Supplementary material for: Comparative performance of SARS-CoV-2 lateral flow antigen tests and association with detection of infectious virus in clinical specimens: a single-centre laboratory evaluation study
Source: Lancet Microbe. 2021 Sep;2(9):e461–71. doi: 10.1016/S2666-5247(21)00143-9 (PMC8245061; doi:10.1016/S2666-5247(21)00143-9)
Supplement: Supplementary appendix [file mmc1.pdf]

THE LANCET
Microbe

Supplementary appendix

This appendix formed part of the original submission and has been peer reviewed. We post it as supplied by the authors.

Supplement to: Pickering S, Batra R, Merrick B. Comparative performance of SARS-CoV-2 lateral flow antigen tests and association with detection of infectious virus in clinical specimens: a single-centre laboratory evaluation study. *Lancet Microbe* 2021; published online June 30. https://doi.org/10.1016/S2666-5247(21)00143-9.

Appendix

This appendix is part of the original submission.

Supplement to: Pickering S, Batra R, Merrick B, et al. Comparative performance of SARS-CoV-2 lateral flow antigen tests and association with detection of infectious virus in clinical specimens: a laboratory evaluation study.

APPENDIX

Table of contents

1. **Supplementary Methods** 3

    1.1. Ethics
    1.2. Cell lines and viruses
    1.3. Intracellular nucleocapsid staining

2. **Supplementary Tables** 4

    2.1. Supplementary Table 1: Rapid antigen LFD names and manufacturers
    2.2. Supplementary Table 2: McNemar's test analyses performed for the comparison of the sensitivity of three commercial SARS-CoV-2 rapid antigen tests
    2.3. Supplementary Table 3: Baseline clinical details for the five patients with sequential samples

3. **Supplementary Figures** 7

    3.1. Supplementary Figure 1: Rapid Antigen test scoring criteria
    3.2. Supplementary Figure 2: Logistic regression and cumulative sensitivity analyses for six commercial rapid antigen tests
    3.3. Supplementary Figure 3: Analyses of the relationship between infectious virus culture and Cts or viral load

1. Supplementary Methods

Ethics

All studies were performed in accordance with the UK Policy Framework for Health and Social Care Research and with specific research ethics committee approval. In accordance with the ethical approval that supports this research (NHS Health Research Authority and Health and Care Research Wales; REC refererence 20/SC/0310), samples were obtained as part of individuals' routine standard of care and surplus VTM was routinely stored at -80oC by the diagnostic laboratory for future technology evaluations. No samples were collected specifically for this study and no written informed consent was required. All swabs and VTM samples were stored in the Directorate of Infection. Samples for research were retrieved by the direct care team and anonymised before sending to the King's College London laboratories for analysis along with dates of symptom onset and sample collection, and any relevant routine laboratory result obtained from that sample.

Cell lines and viruses

Vero-E6 cells (ATCC CRL 1586™) were used for the propagation of SARS-CoV-2 used for LOD and neutralisation assays, virus titre determination and direct virus growth assays on clinical specimens. Cells were cultured at 37°C and 5% CO_2, in Dulbecco's modified Eagle's medium (DMEM, GibcoTM, Thermo Fischer, UK) supplemented with 10% foetal bovine serum (FBS, Merck, Germany).

The UK SARS-CoV-2 reference strain (England/02/2020) was obtained from Public Health England and propagated in Vero-E6 cells. 100µl of reference virus was added to $6x10^6$ Vero-E6 cells and incubated until cell cytopathic effect was observed, at which point cell culture medium was harvested, filtered and aliquoted at -80°C until future use. To determine viral titres as PFUs, virus was 10-fold serially diluted and applied to Vero-E6 cells in 6-well plates, in a volume of 500µl per well, and incubated for 1 hour at 37°C. 500µl of pre-warmed overlay (0.1% agarose in DMEM supplemented with 2% FBS, pen/strep and amphotericin B) was then applied to each well, and cultures were incubated for 72 hours at 37°C, before fixing with 4% formaldehyde. A solution of 0.05% crystal violet (Sigma, Germany) in ethanol was applied to each well, incubated for 5 minutes at room temperature, before washing with PBS, air drying and counting plaques. Plaque assays for the direct measurement of viral titre in clinical samples were done as detailed above, but in 12-well plates.

To determine the association between PFU/mL and N Ct result, RNA was extracted from ten-fold serial dilutions of Vero-E6-titred SARS-CoV-2 (England 02/2020; 10^0 to 10^7 PFU/mL) and assayed by N RT-PCR. The mean Ct for each virus concentration was obtained from three independent experiments.

All work with SARS-CoV-2 England/02/2020, as well as isolation and propagation of viral isolates from swabs, was conducted inside a class II microbiological safety cabinet in a biosafety level 3 (BSL3) facility at King's College London.

Intracellular nucleocapsid staining

Immunostaining for SARS-CoV-2 nucleocapsid detection in Vero.E6 cells was performed in situ in formaldehyde-fixed 96-well plates, to verify viral culture experiments, as described previously[15], sample volume permitting (n=110 of the 141 samples). Briefly, cells were permeabilised with 0.1% triton in PBS for 15 minutes, then blocked in 3% milk for 15 minutes at room temperature. Primary antibody (murinized anti-N 3009[16]) was incubated at a final concentration of 2 µg/mL in 1% milk for 45 minutes at room temperature, before washing twice with PBS and incubating with secondary antibody (goat-anti-mouse IgG HRP-linked, Cell Signaling Technology, 1:2000) in 1% milk for 45 minutes at room temperature. Cells were washed twice with PBS, before addition of substrate. For SARS-CoV-2 plaque verification assays, TrueBlue HRP substrate was used (Seracare Life Sciences Inc.).

2. Supplementary Tables

Supplementary Table 1: Rapid antigen LFDs

Details of each rapid antigen LFD used in this study are given, with the name referred to throughout the study in the left-hand column. Full commercial names and manufacturers are also given, alongside viral target (if disclosed by the manufacturer) and the manufacturer's own sensitivity and specificity determinations.

Rapid Antigen LFD	Commercial Name	Manufacturer/ Distributor	Reference	Viral target*	Characteristics according to Manufacturer		
					Sensitivity**	Specificity	LoD
Innova	Rapid SARS-CoV-2 Antigen Test	Xiamen Biotime Biotechnology, Ltd (Fujian, China) / Innova Medical Group (Pasadena, California, USA)	N/A	Nucleocapsid	96%	100%	4.25 x 10^{2} TCID50/ml
Spring Healthcare	SARS-CoV-2 Antigen Rapid Test Cassette (Swab)	Shanghai ZJ Bio-Tech Co.,Ltd (Shangai, China) / Spring Healthcare Services AG (Switzerland)	SP-SW 106		84.4%	100%	1.25 x 10^{3} TCID50/ml
E25 Bio	Rapid Diagnostic Test	E25Bio (Cambridge, Massachusetts, USA)	N/A	Nucleocapsid	84.7%	85.7%	N/A
Encode	SARS-CoV-2 Antigen Rapid Test Device	Encode (Zhuhai, China) / Emmo Pharma (Macclesfield, UK)	N/A	Nucleocapsid	86.7%	100%	N/A
SureScreen F	COVID-19 Rapid Fluorescent Antigen Test	SureScreen Diagnostics Ltd (Derby, UK)	COVID19 AGC	Nucleocapsid	92.9%	98.6%	N/A
SureScreen V	COVID-19 Rapid Antigen Test Cassette (Nasopharyngeal Swab)	SureScreen Diagnostics Ltd (Derby, UK)	COVID19 AGVCT	Nucleocapsid	94.5%	99.9%	2.0 x $10^{2.4}$ TCID50/ml

* Note that this information was not available for all tests.
** The Ct cut-offs for the samples used on these evaluations were not disclosed in the inserts and/or clinical validation reports accompanying the Rapid Antigen tests.

Supplementary Table 2: McNemar's test analyses performed for the comparison of the sensitivity of three commercial SARS-CoV-2 rapid antigen tests.

Comparison of the sensitivities of Innova, Encode and SureScreen F were performed using McNemar's test, using the subset of samples shown in Table 2. Two-tailed *p*-values and Chi-square values are shown for all permutations.

		Total (N)	p-value	Chi-square
Innova v Encode	Overall	90	0.7237 (ns)	0.125
	Ct < 28	70	0.6831 (ns)	0.167
	Ct < 25	52	1.000 (ns)	0.000
Innova v SureScreen F	Overall	90	0.0001 (***)	15.429
	Ct < 28	70	0.0012 (**)	10.563
	Ct < 25	52	0.0044 (**)	8.100
Encode v SureScreen F	Overall	90	0.0001 (***)	15.059
	Ct < 28	70	0.0015 (**)	10.083
	Ct < 25	52	0.0077 (**)	7.111

Supplementary Table 3: Baseline clinical details for the five patients with sequential samples.

	Age	Sex	Ethnicity	Comorbidities	Disease Severity	Reason for Hospitalisation
**Patient 1**	57	F	Black British	Hypertension, pancreatic cancer	0	Acute kidney injury
**Patient 2**	79	M	White British	Hypertension, IgA nephropathy, ischemic heart disease	0	Non-ST elevation myocardial infarction
**Patient 3**	50	F	Black African	End-stage renal failure, renal transplant, obesity, type-2 diabetes	4	COVID-19
**Patient 4**	69	F	N/A	Immune thrombocytopenia	1	Immune thrombocytopenia
**Patient 5**	74	M	White British	Rectal cancer	1	Elective surgical admission

Patients that tested positive by RT-PCR for SARS-CoV-2 were classified for COVID-19 severity as follows[15,16]:

0- asymptomatic OR no requirement for supplemental oxygen
1- requirement for supplemental oxygen (FiO_2 <0.4) for at least 12 hrs.
2- requirement for supplemental oxygen (FiO_2 ≥0.4) for at least 12 hrs.
3- requirement for non-invasive ventilation (NIV)/ continuous positive airways pressure (CPAP) OR proning OR supplemental oxygen (FiO_2 >0.6) for at least 12 hrs AND not a candidate for escalation above level one (ward-based) care.
4- requirement for intubation and mechanical ventilation OR supplemental oxygen (FiO_2 >0.8) AND peripheral oxygen saturations <90% (with no history of type 2 respiratory failure (T2RF)) OR <85% (with known T2RF) for at least 12 hrs.
5- requirement for extracorporeal membrane oxygenation (ECMO).

Severity scores of 1-3 correspond to mild COVID-19, while scores of 4-5 correspond to severe COVID-19.

Supplementary Figure 1: Rapid antigen test scoring criteria

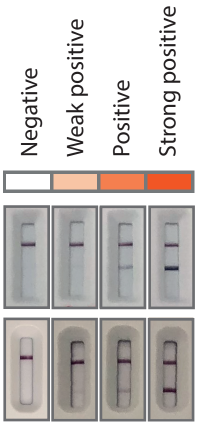

Visual examples of the rapid antigen test scoring criteria for band classifications: 0 (negative), 0.5 (weak positive), 1 (positive) and 2 (strong positive). Examples are shown for two different tests. Note that bands scored as 0.5 are visible on the test cassette but often do not appear clearly positive in photographs.

Supplementary Figure 2: Logistic regression and cumulative sensitivity analyses for six commercial rapid antigen tests

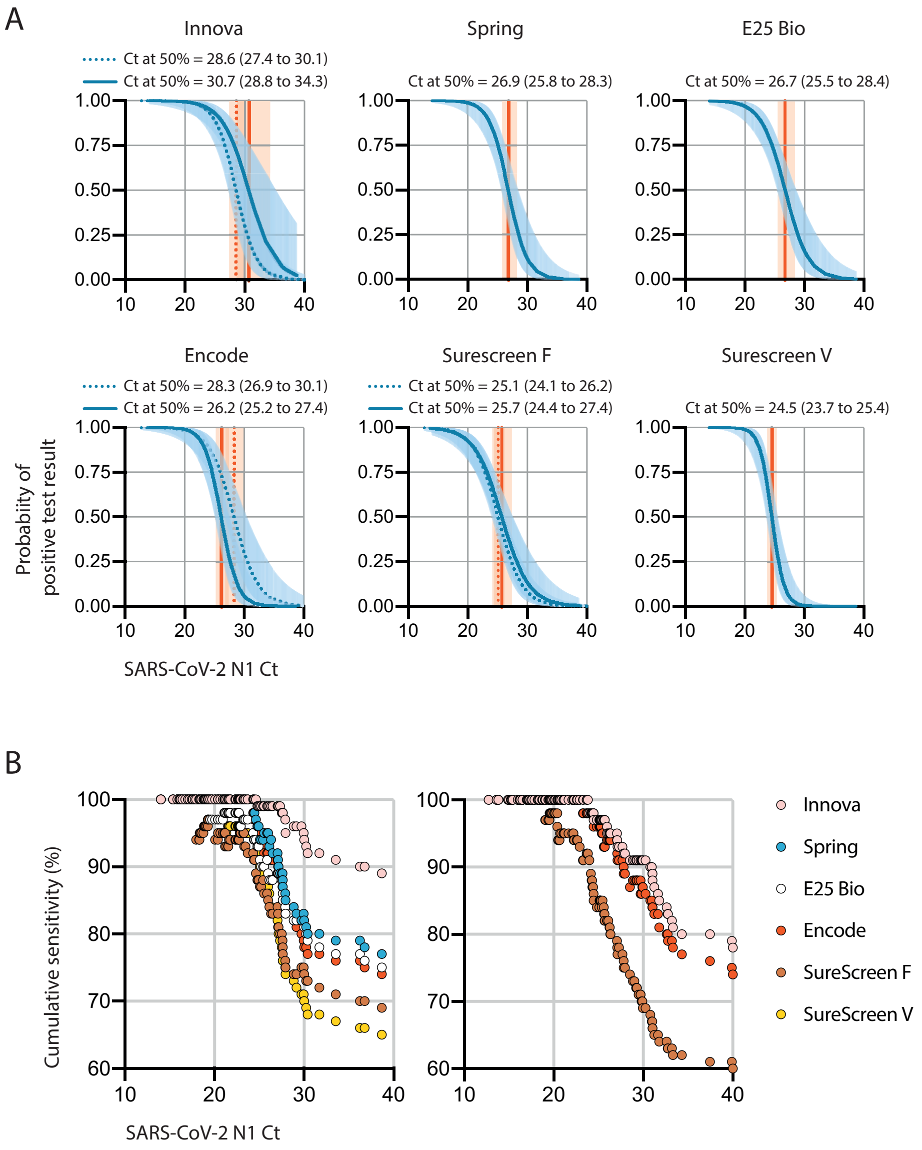

(A) Logistic regression analyses for the sensitivity data presented in Figure 1D, using an identical panel of 100 SARS-CoV-2 positive swab samples on six commercial rapid antigen tests. Logistic plots are shown in blue, with light blue shaded areas representing 95% CIs. Vertical red lines indicate the predicted Ct value at which 50% of the samples gave a positive result, with shaded area indicating 95% CIs; these values are indicated above each plot. For Innova, Encode and SureScreen F, logistic regression analyses are also included for the data presented in Figure 2A, shown as dashed lines, with shaded areas indicating 95% CIs. Areas of darker shading indicate regions at which 95% CIs overlap. The further to the right the line is, the better the overall sensitivity performance across a range of Ct values; a higher 'Ct at 50%' value corresponds to a more sensitive test.

(B) Cumulative sensitivity determinations for the data presented in Figure 1D (left panel) and Figure 2A (right panel). Test sensitivities, determined relative to RT-PCR result, were calculated cumulatively for ascending Cts, and plotted as a function of increasing Ct.

Supplementary Figure 3: Analyses of the relationship between infectious virus culture and Ct or viral load

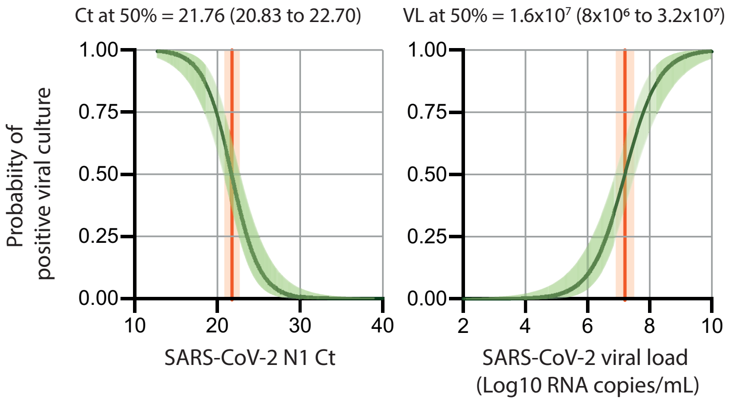

Logistic regression analyses for the data presented in Figure 2A, showing the probability of positive viral culture according to Ct value (left panel) or SARS-CoV-2 viral load (log10 RNA copies/mL, right panel) on 138 SARS-CoV-2 positive swabs. The three SARS-CoV-2 positive swabs which growth was indetermined are not included. Logistic plots are shown in green, with light green shaded areas representing 95% CIs. The predicted Ct value and viral load at which 50% of the cultures were positive are shown as vertical red lines, with shaded area indicating 95% CIs; these values are indicated above each plot.
